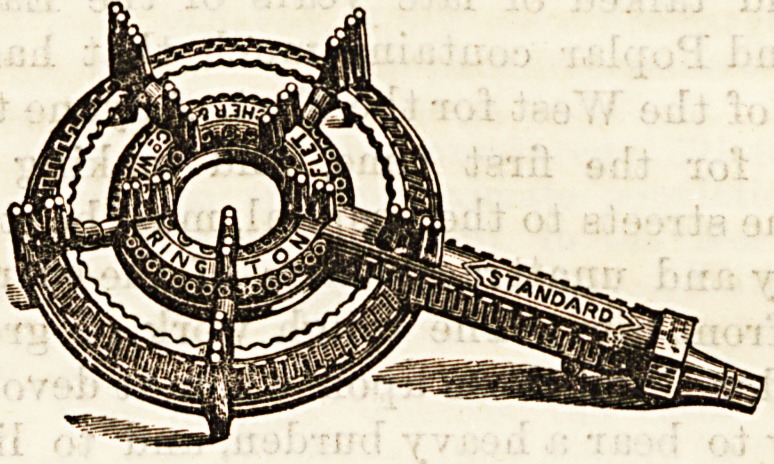# Gas Stove

**Published:** 1893-10-28

**Authors:** 


### PRACTICAL DEPARTMENTS.

### GAS STOVE.

We have previously, in these columns, gone exhaustively into the subject of cooking by gas, and fully enumerated the many advantages which are to be derived from its use, both in institutions and in private houses. (*See* The Hospital for January 7th, 14th, 21st, and 28th, 1893.) We have received a small burner, of which an illustration is given below, from Messrs. Fletcher, Russell, and Co., of Warrington. We have commented before on the comfort these simple stoves prove in the sick-room, obviating noise, in summer doing away with the necessity for a fire, and being quickly and easily put into working order; while with the aid of a good sized, flat-bottomed kettle, a plentiful supply of boiling water can be had at very few minutes' notice. A simple indiarubber tube, which is readily adjustable, carries the gas supply from the ordinary burner to the little stove, which, as can be seen, consists of a pierced metal circle, with a fixed arrangement above for the support of kettle or saucepan. The special advantage claimed for Messrs. Fletcher and Russell's stove over others of the same make and size lies in the new form of enamel with which it has been treated. By this latest process all danger of rust is done away, and the enamel may be had in any colour which may be preferred, no amount of heat causing any change of tint. The coat of paint is extremely thin, but quite permanent, and impervious to dirt and smoke. These little burners are very inexpensive, and we warmly recommend them to all, confident that when they have once received a trial they will be universally acknowledged to be productive of much saving of time and trouble, while economy is undoubtedly not the least amongst their good points. Our illustration is by kind permission of the manufacturers.

## Figures and Tables

**Figure f1:**